# Metabolites of nitric oxide as a marker cardiometabolic in blacks

**DOI:** 10.1186/1758-5996-7-S1-A157

**Published:** 2015-11-11

**Authors:** Patricia Maurer, Jamila Benvegnu Bruno, Angelica Aparecida da Costa Gullich, Patricia Martinez Oliveira, Bruna Cocco Pilar, Ritiele Pinto Coelho, Vinicius Tejada Nunes, Vanusa Manfredini, Jacqueline da Costa Escobar Piccoli

**Affiliations:** 1 Universidade Federal do Pampa, Uruguaiana, Brazil

## Background

The black population has a high cardiometabolic risk, however low incidence of metabolic syndrome (MetS). In this context, there seems to be a paradox related to the diagnostic criteria of MetS, which makes the presence of the same is underestimated in blacks. This condition brings the need to look for a more reliable markers of actual pathological conditions and cardiometabolic risk of these individuals. A possible marker is nitric oxide (NO). The dosage metabolites nitrite/nitrate (NOx) have been shown to be associated with some criteria of the metabolic syndrome, such as obesity and diabetes, however the literature lacks more specific studies to assess whether altered levels of nitric oxide are associated with metabolic and cardiovascular disease among blacks.

## Aim

Analyze the NOx levels as a marker of cardiometabolic alterations in Brazilian blacks, and their applicability as a biomarker of cardiometabolic risk.

## Materials and methods

Several anthropometric, biochemical, inflammatory, oxidative and hematological parameters and their relationship with nitric oxide metabolites were measured. NOx levels were distributed in percentiles, the 50% percentile=122.3μmol/L was chosen as the cutoff point.

## Results

The study included 202 self-declared black people, mostly women, average age 45 yrs. old. Anthropometric assessment showed that most of the study population had an average BMI classified as grade 1 obesity and altered waist circumference. The MetS was found in 61% of the sample, hypertension in 53% and diabetes in 13.5% of black subjects. NOx values <122.3 µmol/L were associated with higher body mass index (p=0.01), waist circumference (p=0.03) and hip circumference (p=0.04). As to biochemical criteria, the NOx was significantly correlated to blood glucose levels (p=0.04), triglycerides (p=0.04), albumin (p=0.03), uric acid (p=0.01) and urea (p=0.05). In inflammatory and oxidative stress assessment, only protein carbonylation (p<0.01) was associated with the NOx, in which the damage was greater in subjects who had NOx values lower than the 50% percentile. There was no association between NOx and the blood profile.

## Conclusion

To be mainly related to obesity, dyslipidemia and glucose, the NOx was considered a good predictor of cardiometabolic risk in black population, besides being easily adapted to laboratory testing and routine and can be programmed for automatic or semi-automated analyzers, thus requiring very little sample preparation.

